# Demographic Differences and Associations Between Body Image and Quality of Life Among Japanese Adolescents with Congenital Physical Disabilities

**DOI:** 10.3390/bs14121209

**Published:** 2024-12-17

**Authors:** Minkyeong Kim, Kennosuke Kawama, Yongjae Lim

**Affiliations:** 1 Graduate School of Comprehensive Human Sciences, University of Tsukuba, Tsukuba 305-8577, Japan; 2 Institute of Human Science, University of Tsukuba, Tsukuba 305-8577, Japan; kawama@human.tsukuba.ac.jp; 3 Faculty of Education, Chiba University, Chiba 263-8522, Japan; im@chiba-u.jp

**Keywords:** body image, quality of life, adolescent, physical disability, Japan

## Abstract

This study aimed to examine the levels, demographic differences, and associations between body image (BI) and quality of life (QOL) among Japanese adolescents with congenital physical disabilities. A self-report questionnaire was administered to 107 Japanese adolescents with congenital physical disabilities, which included demographic variables, the Multidimensional Body-Self Relations Questionnaire, and the Japanese version of the World Health Organization Quality of Life Brief Version. Data were analyzed using Cronbach’s α coefficient, descriptive statistics, *t*-tests, one-way ANOVA, and Pearson’s correlation coefficient. The mean BI score was 2.96 ± 0.39. The domain-specific mean scores were as follows: health evaluation (3.49 ± 0.67), health orientation (3.11 ± 0.57), fitness evaluation (3.03 ± 0.93), fitness orientation (3.02 ± 0.70), appearance orientation (2.70 ± 0.61), and appearance evaluation (2.61 ± 0.61). The mean QOL score was 3.51 ± 0.50. The domain-specific mean scores were as follows: social relationships (3.61 ± 0.91), environment (3.60 ± 0.59), physical (3.46 ± 0.55), and psychological (3.42 ± 0.63). BI significantly varied by gender, school level, type of disability, and activities of daily living (ADLs), while QOL varied by school level and ADLs. All BI domains, except appearance orientation (investments for enhancing appearance), were positively correlated with QOL. Our findings suggest that school level and ADLs are key predictors of both BI and QOL among Japanese adolescents with congenital physical disabilities and that physical-fitness- and health-related BI are closely associated with QOL.

## 1. Introduction

A physical disability is a condition that limits or impairs an individual’s physical functioning in one or more limbs, either temporarily or permanently [1]. The causes of physical disabilities are diverse, including congenital factors such as hereditary conditions, and acquired factors such as accidents, injuries, illnesses, and post-surgical effects [2]. Physical disabilities significantly limit activities of daily living (ADLs), such as eating, dressing, bathing, and mobility. Additionally, they may contribute to psychological issues by increasing emotional, social, and behavioral symptoms [3,4]. These psychological issues can lead to a decline in body image (BI) and quality of life (QOL) among individuals with physical disabilities [5], with potentially greater effects on adolescents who are undergoing significant physical and psychological growth and development during puberty.

BI is a complex and multidimensional construct encompassing an individual’s perceptions and attitudes toward physical appearance, bodily structure, and functional capabilities [6,7]. It includes aspects of physical appearance, fitness, and health [8]. A negative BI can lead to lower self-esteem [9], depression [10], social isolation [11], and eating disorders [12]. In the field of physical disabilities, most research on BI has primarily concentrated on individuals with acquired physical disabilities, particularly adults, and has consistently shown that such disabilities adversely affect their BI. However, research focusing on adolescents with congenital physical disabilities is limited, and findings regarding the relationship between physical disabilities and BI remain inconsistent. For instance, Kim et al. [13] found that adolescents with congenital physical disabilities tend to have a predominantly positive BI. Their study did not find any significant differences in BI based on the type of disability, specifically when comparing the cerebral palsy group to other physical disability groups. In contrast, Hammar et al. [14] reported that the BI of adolescents with cerebral palsy is less favorable than that of their peers. These findings highlight the need for foundational research on the BI of adolescents with congenital physical disabilities.

QOL is “an individual’s perception of their life situation within their cultural and value systems, considering their goals, expectations, standards, and concerns” [15]. It is a multidimensional construct encompassing physical, psychological, social, and environmental domains of well-being. QOL is frequently used as a key indicator in healthcare and social science research to assess an individual’s overall life satisfaction and well-being [16]. In the field of physical disabilities, research on QOL has predominantly focused on adults with acquired physical disabilities, often reporting that such conditions are closely associated with lower QOL. Research on the QOL of adolescents with congenital physical disabilities remains limited [17], and the generalizability of existing findings is constrained. Some studies have indicated that the QOL of adolescents with congenital physical disabilities is often comparable to, or even higher than, that of their peers without disabilities [18,19,20]. However, other studies have reported that adolescents with cerebral palsy—a common congenital physical disability—exhibit lower QOL compared to their typically developing peers [21,22]. These inconsistencies underscore the need for more foundational research on the QOL of adolescents with congenital physical disabilities.

Recent studies have increasingly investigated BI and QOL simultaneously, particularly among adult patients and female patients who have experienced bodily changes due to specific events such as breast cancer [23], obesity [24], and amputations [25,26]. In recent years, researchers in healthcare and psychology have recognized the need to consider both BI and QOL to understand patients’ overall well-being. Additionally, there has been a growing trend toward patient-centered research that emphasizes individual experiences and perceptions, contributing to the rise in studies examining both BI and QOL together. As efforts to evaluate and enhance patients’ QOL have expanded, research into the relationship between these concepts has become more prevalent. Most studies have reported that BI and QOL in this patient group are generally lower than those in the control group, with a strong relationship between the two concepts [26,27]. For instance, Sarroca et al. [26] reported that the physical changes caused by amputation significantly affect physical, functional, and emotional aspects, negatively impacting the BI and QOL of individuals with amputations. Research has established that bodily changes due to acquired conditions (e.g., diseases or accidents) directly influence an individual’s psychological state, including BI and QOL [25,28]. Few studies have specifically examined the levels and associations between BI and QOL among adolescents with congenital physical disabilities. Due to the limited scope of prior research, drawing definitive conclusions remains challenging. However, BI and QOL tend to be higher in males and younger adolescents with congenital physical disabilities [13,29,30,31], while differences based on the type of disability and ADLs remain inconsistent [13,17,32]. Particularly regarding ADLs, studies have presented mixed findings: some report that adolescents with severe limitations have higher perceptions of BI or QOL [13,33], while others suggest that those with mild limitations exhibit greater satisfaction with BI or QOL [34]. Consequently, comprehensive foundational research is necessary to elucidate perceived BI and QOL, including examining differences in their levels based on factors such as gender, school level, type of disability, and ADLs, as well as exploring the associations between these two concepts. The study findings will provide foundational data for psychological research on BI and QOL in adolescents with congenital physical disabilities. These findings will guide the development of educational and intervention strategies to promote a positive BI and enhance QOL.

The primary aims of this study were as follow:

1. To investigate the levels of BI and QOL among Japanese adolescents with congenital physical disabilities;
2. To identify whether there are differences in BI and QOL of adolescents with congenital physical disabilities in relation to demographic variables such as gender, school level, type of disability, and ADLs;
3. To determine whether BI and QOL are associated in adolescents with congenital physical disabilities.

## 2. Materials and Methods

### 2.1. Study Design and Participants

This cross-sectional, school-based study was conducted in Japan between June and July 2022. Data were collected from adolescents with congenital physical disabilities across the country using a self-administered paper-based questionnaire. The study focused on adolescents with congenital physical disabilities who met the following inclusion criteria: (1) enrolled in grades 4 to 12 (ages 10 to 18 years); (2) without intellectual, visual, or hearing impairments, or any other coexisting disabilities; and (3) capable of independently reading and comprehending the questionnaire.

In 2021, Japan had 118 schools for students with physical disabilities [35]. For this study, 59 schools were randomly selected, in consideration of the number of schools the research team could manage within the two-month study period. The study’s purpose, methods, and ethical considerations were explained to these schools, and questionnaires were provided to the 38 schools where eligible students agreed to participate. A total of 135 questionnaires were provided to adolescents with congenital physical disabilities who met the inclusion criteria. The study was approved by the Institutional Review Board of the University of Tsukuba (Tsukuba 2022-31A).

### 2.2. Measures

The dependent variables in this study were BI and QOL. BI was assessed using the Multidimensional Body-Self Relations Questionnaire (MBSRQ) [8] and QOL was evaluated using the Japanese version of the World Health Organization Quality of Life Brief Version (WHOQOL-BREF) [36]. The independent variables included gender, school level, type of disability, and ADLs. To examine the associations between the domains of BI and QOL, these domains were used as analysis variables.

#### 2.2.1. Demographic Variables

Demographic variables included gender (male and female), school level, type of disability, and ADLs. School level was categorized into elementary school (10–12 years), middle school (13–15 years), and high school (16–18 years). The type of disability was divided into cerebral palsy and others (e.g., muscular dystrophy, osteogenesis imperfecta, and arthrogryposis multiplex congenita). ADLs were assessed based on five tasks: eating, dressing, bathing, toileting, and moving. Each activity was scored on a scale ranging from 1 (able to perform independently), 2 (requiring some assistance), to 3 (requiring total assistance) [37]. The total scores ranged from 5 to 15, with higher scores indicating greater assistance needs. A score of 5 indicates mild limitations, 6 to 10 indicates moderate limitations, and 11 to 15 indicates severe limitations in ADLs.

#### 2.2.2. Body Image

BI was assessed using Cash’s [8] Multidimensional Body-Self Relations Questionnaire (MBSRQ). This measure is widely used in several countries, including Japan, and comprises 10 domains and 69 items. Researchers often choose certain domains for the tool depending on the purpose of their study [8]. In this study, 49 items for six domains were used. Appearance evaluation (7 items) measures feelings of physical attractiveness or unattractiveness and satisfaction or dissatisfaction with one’s look; appearance orientation (12 items) measures the extent of investment in one’s appearance; fitness evaluation (3 items) measures feelings of being physically fit or unfit; fitness orientation (13 items) measures the extent of investment in being physically fit; health evaluation (6 items) measures feelings of physical health and/or freedom from illness; and health orientation (8 items) measures the extent of investment in a physically healthy lifestyle. Each item was rated on a five-point Likert scale from 1 (definitely disagree) to 5 (definitely agree), and a higher score indicated greater BI. The Cronbach’s α was 0.73–0.94 in the study by Cash [8] and 0.66–0.89 in this study.

#### 2.2.3. Quality of Life

The Japanese version of the World Health Organization Quality of Life Brief Version (WHOQOL-BREF) was used [36]. The WHOQOL-BREF comprises two questions addressing overall QOL and general health QOL. It contains 24 items divided into four domains: physical (7 items), psychological (6 items), social relationships (3 items), and environment (8 items). Each item is rated on a five-point Likert scale from 1 (very dissatisfied/not at all) to 5 (very satisfied/extremely), with a higher score indicating better QOL. The WHOQOL-BREF is widely used in several countries, including Japan, and has been demonstrated to have consistent reliability and validity [38,39]. The Cronbach’s α was 0.66–0.82 in the Japanese version of the WHOQOL-BREF [36] and 0.63–0.90 in this study.

### 2.3. Data Analysis

Data analysis was performed using SPSS Statistics Version 27.0 [40]. Missing data were handled according to the guidelines for each measurement. Participants with excessive missing data that could not be addressed under these guidelines were excluded from the analysis. The reliability of the measures (MBSRQ and WHOQOL-BREF) was assessed using Cronbach’s α coefficients for the study participants.

Descriptive statistics were calculated for demographic characteristics (gender, school level, type of disability, and ADLs) and key study variables, including total and domain-specific levels of BI and QOL.

The *t*-test and one-way ANOVA were conducted to identify between-group comparisons. These analyses aimed to determine whether the differences observed for each dependent variable were statistically significant based on the various independent variables. Specifically, the *t*-test was used to compare the means of two groups (gender and type of disability), and the one-way ANOVA was used to compare the means of three groups (school level and ADLs). When a significant difference was found in the one-way ANOVA, post hoc Scheffé’s tests were performed to identify significant differences between groups.

Pearson correlation coefficients were calculated to investigate the possible significant associations between the two dependent variables, BI and QOL domains. Effect sizes were calculated to assess the practical significance of our findings, following Cohen’s [41] guidelines for interpretation. The level of statistical significance was set at a *p* value of less than 0.05. 

## 3. Results

### 3.1. Demographic Characteristics

A total of 135 questionnaires were distributed, resulting in responses from 110 participants (response rate: 81.48%); of these, 3 were excluded due to incomplete data.

Demographic characteristics are shown in Table 1. A total of 107 participants were included in the analysis, with 54 (50.5%) male and 53 (49.5%) female participants. The participants’ mean age was 14.6 years ± 2.28 (median = 15), ranging from 10 to 18 years. The distribution by school level was as follows: elementary school (N = 22, 20.6%), middle school (N = 45, 42.1%), and high school (N = 40, 37.4%). Fifty-two (48.6%) had cerebral palsy, while fifty-five (51.4%) had other physical disabilities. Regarding ADLs, the mean score was 8.08 (*SD* = 3.04, range = 5–15, median = 8). The distribution by ADLs was as follows: 38 participants had mild limitations (35.5%), 42 had moderate limitations (39.3%), and 28 had severe limitations (25.2%).

### 3.2. Scores of Body Image and Quality of Life

The scores of BI and QOL are shown in Table 2. The mean BI score was 2.96 (*SD* = 0.39). By domain, the mean scores were as follows: health evaluation (*M* = 3.49, *SD* = 0.67), health orientation (*M* = 3.26, *SD* = 0.64), fitness evaluation (*M* = 3.03, *SD* = 0.93), fitness orientation (*M* =3.02, *SD* = 0.70), appearance orientation (*M* = 2.70, *SD* = 0.61), and appearance evaluation (*M* = 2.61, *SD* = 0.61). The mean QOL score was 3.51 (*SD* = 0.50). By domain, the mean scores were as follows: social relationships (*M* = 3.61, *SD* = 0.91), environment (*M* = 3.60, *SD* = 0.59), physical (*M* = 3.46, *SD* = 0.55), and psychological (*M* = 3.42, *SD* = 0.63).

### 3.3. Differences in Body Image and Quality of Life According to Demographic Variables

The differences in BI according to demographic variables are shown in Table 3.

First, the mean BI score significantly differed according to ADLs. Adolescents with mild limitations had higher BI than those with severe limitations (*F* = 4.46, *p* < 0.05, effect size = 0.08). Next, each of the BI domains significantly differed according to gender, school level, type of disability, and ADLs. In terms of gender, female participants had higher appearance orientation than male participants (*t* = −2.40, *p* < 0.05, effect size = −0.46), while male participants had higher fitness evaluation (*t* = 2.23, *p* < 0.05, effect size = 0.43) and fitness orientation (*t* = 3.04, *p* < 0.01, effect size = 0.59) than female participants. In terms of school level, elementary school students had higher fitness evaluation than high school students (*F* = 5.69, *p* < 0.01, effect size = 0.10), while middle school students had higher fitness orientation than high school students (*F* = 6.02, *p* < 0.01, effect size = 0.10). In terms of type of disability, those with other physical disabilities had higher fitness evaluation than those with cerebral palsy (*t* = −2.75, *p* < 0.01, effect size = −0.53). In terms of ADLs, those with mild limitations had higher appearance orientation than those with severe limitations (*F* = 3.32, *p* < 0.05, effect size = 0.06), and those with mild and moderate limitations had higher fitness evaluation (*F* = 13.76, *p* < 0.001, effect size = 0.21) and fitness orientation (*F* = 6.15, *p* < 0.01, effect size = 0.11) than those with severe limitations.

The differences in QOL according to demographic variables are shown in Table 4. The overall QOL score did not significantly differ according to demographic variables. However, the QOL domains significantly differed according to school level and ADLs. In terms of school level, middle school students had higher psychological domains than high school students (*F* = 3.93, *p* < 0.05, effect size = 0.07). In terms of ADLs, participants with severe limitations had higher psychological domains than those with moderate limitations (*F* = 4.60, *p* < 0.05, effect size = 0.08). In comparison, participants with moderate and severe limitations had higher social relationship domains than those with mild limitations (*F* = 6.03, *p* < 0.01, effect size = 0.10).

### 3.4. Correlations Between Body Image and Quality of Life

The correlations between BI and QOL are shown in Table 5. Significant positive correlations were observed between all BI domains and QOL, except for appearance orientation. By domain, appearance evaluation was significantly positively correlated with the psychological domain (*r* = 0.42, *p* < 0.001) and the social relationship domain (*r* = 0.24, *p* < 0.05). Fitness evaluation was significantly positively correlated with all domains of QOL. Fitness orientation was significantly positively correlated with the physical domain (*r* = 0.25, *p* < 0.05) and the environment domain (*r* = 0.24, *p* < 0.05). Health evaluation was significantly positively correlated with the physical domain (*r* = 0.39, *p* < 0.001), the social relationship domain (*r* = 0.38, *p* < 0.001), and the environment domain (*r* = 0.34, *p* < 0.001). Health orientation was significantly positively correlated with the physical domain (*r* = 0.31, *p* < 0.01), the psychological domain (*r* = 0.25, *p* < 0.05), and the environment domain (*r* = 0.27, *p* < 0.01). No significant correlations were found between appearance orientation and the domains of QOL.

## 4. Discussion

Research on BI and QOL is increasing across various populations, with growing attention given to the associations between these concepts. However, little research has focused on both BI and QOL among Japanese adolescents with congenital physical disabilities. Therefore, this study is significant as the first investigation into the levels, demographic differences, and associations between BI and QOL among Japanese adolescents with congenital physical disabilities.

The first aim of this study was to investigate the levels of BI and QOL among participants. The level of BI in this study was found to be comparable to that of Korean adolescents with congenital physical disabilities [13], as well as to peers without disabilities in Korea [42] and Taiwan [43]. This indicates that having a congenital disability does not always result in lower BI, contrasting with previous studies that reported lower BI among individuals with acquired disabilities compared to the control group [25,44]. These findings indicate that the BI of individuals with congenital disabilities should be assessed using distinct criteria from those applied to individuals with acquired disabilities.

Health evaluation was the highest by domain, followed by health orientation, fitness evaluation, fitness orientation, appearance orientation, and appearance evaluation. This finding indicated that these adolescents were generally more satisfied with their physical fitness and health than with their appearance. This aligns with research conducted on adolescents with congenital physical disabilities in Korea [13]. Notably, when compared with studies using the MBSRQ, the level of appearance evaluation and appearance orientation among Japanese and Korean adolescents with congenital physical disabilities was not lower than that of non-disabled middle school students in Korea [13,42] and non-disabled junior high school students in Taiwan [43], with some domains even showing slightly higher scores. At the same time, it cannot be conclusively determined that congenital physical disabilities directly cause a decrease in appearance-related BI. Adolescents with and without disabilities tend to evaluate their appearance negatively in predominantly Asian countries such as Japan, Korea, and Taiwan [45,46]. Hung et al. [47] noted that Asian adolescents are generally at a higher risk of experiencing dissatisfaction with their appearance-related BI. This tendency is influenced by dissatisfaction with their overall body conditions, concerns about specific body parts, and worries about being overweight.

The level of QOL in this study demonstrated balanced satisfaction across all domains, which is similar to the findings in Korean adolescents with congenital physical disabilities [13], whose QOL was reported as not poor. Due to the limited research using the WHOQOL-BREF for direct comparison with a control group, comparisons are challenging. This suggests that the QOL of adolescents with congenital physical disabilities in Japan and Korea may be comparable to or even higher than that of their peers without disabilities. While different scales were used, Kato et al. [19] found no significant differences in QOL between adolescents with congenital physical disabilities and the control group. This contrasts with studies reporting lower QOL in individuals with acquired disabilities compared to the control group [48,49], indicating that the QOL assessment for individuals with congenital disabilities requires a distinct approach.

Social relationships ranked the highest by domain, followed by the environment, physical, and psychological domains. Although the differences were not significant, Japanese adolescents with congenital physical disabilities reported greater satisfaction with social relationships compared to other domains. In contrast, adolescents with physical disabilities in Malaysia [50] and those with cerebral palsy in Slovenia [51] showed the least satisfaction with social relationships when compared to other aspects of their QOL. Japanese adolescents with congenital physical disabilities appear to encounter fewer challenges related to social relationships compared to their peers in other countries. This may indicate that Japan’s educational environment and social support system function well and effectively accommodate the needs of adolescents with congenital physical disabilities [19].

The second aim of this study was to identify differences in BI and QOL according to demographic variables. BI significantly differed by gender, school level, type of disability, and ADLs, while QOL significantly differed by school level and ADLs. The effect size showed a small effect only for gender, while all other demographic variables demonstrated medium or large effect sizes, indicating practical importance. School level and ADLs emerged as common predictors for both BI and QOL. Elementary and middle school students reported a higher perception of fitness-related BI than high school students, and middle school students exhibited higher psychological QOL than high school students. These findings are consistent with previous studies indicating that BI and QOL tend to decrease with age, regardless of disability status [13,52]. This decline in BI and QOL with age suggests a general adolescent trend.

Adolescents with congenital physical disabilities who had mild ADL limitations reported a higher perception of BI in appearance orientation, fitness evaluation, and fitness orientation compared to those with moderate or severe ADL limitations. This may be attributed to the greater ability of adolescents with mild limitations to manage their appearance independently and engage in physical exercise more easily, allowing them to invest more time and effort in their appearance and fitness. However, previous studies have reported conflicting results [34,53]. For example, Korean adolescents with congenital physical disabilities showed no significant difference in appearance orientation, but those with severe limitations reported higher satisfaction with their appearance (appearance evaluation) compared to those with moderate limitations [13]. While fitness evaluation aligned with the findings of this study, no significant differences were found in fitness orientation. A comprehensive review of studies on congenital physical disabilities in Japan and Korea [13] indicates that the relationship between ADLs and BI lacks consistency, and the limited number of studies complicates the identification of discernible patterns.

Adolescents with congenital physical disabilities who had severe ADL limitations reported higher scores in the psychological domains of QOL than those with moderate limitations, and those with moderate or severe ADL limitations showed higher scores in the social relationship domains of QOL than those with mild limitations. Notably, Japanese adolescents with congenital physical disabilities who had severe ADLs demonstrated a higher QOL compared to their peers with mild ADLs. This finding aligns with previous research on the QOL of adolescents with cerebral palsy [19] and those with neuromuscular disease [54]. It suggests that ADL limitations do not necessarily lead to a negative QOL for adolescents with congenital physical disabilities [17]. Jiang et al. [55] reported that ADL limitations affect physical function and well-being in adolescents with cerebral palsy but are not related to their mental and psychological QOL. This may reflect the widespread acceptance of the ideology of independent living in Japanese society. This ideology posits that “independence” encompasses more than just the ability to perform ADLs autonomously—it also includes the capacity to make critical life decisions independently and to live a fulfilling life with the support of family, friends, and welfare services, even if one is unable to perform ADLs independently [56].

The third aim of this study was to determine the association between BI and QOL among adolescents with congenital physical disabilities. All domains of BI were significantly and positively correlated with QOL. However, appearance orientation was not correlated with any domain of QOL. This finding is consistent with previous research on Korean adolescents with congenital physical disabilities [13]. Integrating the findings from studies conducted in both Japan and Korea indicates that adolescents with congenital physical disabilities demonstrate a stronger relationship between fitness- and health-related BI and QOL compared to appearance. However, appearance orientation was not significantly correlated with any subdomain of QOL. This finding contrasts with previous studies conducted on patients or individuals with acquired disabilities [12,57], which indicated a strong association between appearance-related BI and QOL. Adolescents with congenital physical disabilities may perceive their BI and QOL differently than individuals with acquired disabilities or non-disabled individuals. The results of this study are not confined to adolescents with congenital physical disabilities in Japan; they have been consistently observed in both Japan and Korea. Future research should prioritize fitness- and health-related BI over appearance. Additionally, the low correlation between appearance orientation in BI and QOL among adolescents with congenital physical disabilities warrants further exploration through qualitative studies.

This study has several limitations. First, the questionnaire contained a relatively large number of items, making respondent recruitment challenging and leading to an insufficient sample size. Therefore, caution is necessary when considering the generalizability of the findings. Future studies should focus on recruiting larger samples to enhance the robustness and applicability of the results. Additionally, comparisons between control groups and across countries may offer valuable insights. Second, the cross-sectional design of this study limits the ability to establish temporal or causal relationships between BI and QOL. Longitudinal studies are necessary to provide deeper insights into how these factors evolve over time. Finally, this study primarily focused on collecting fundamental data on BI and QOL among adolescents with congenital physical disabilities, limiting the exploration of more nuanced aspects of these constructs. Future studies should integrate both quantitative and qualitative approaches, such as interviews, to provide a more comprehensive understanding of BI and QOL in this population.

## 5. Conclusions

To the best of our knowledge, this is the first study to examine the levels, demographic differences, and associations between BI and QOL among Japanese adolescents with congenital physical disabilities. The results of this study suggest that adolescents with congenital physical disabilities do not necessarily have lower BI and QOL compared to their peers without disabilities. School level and ADLs emerged as significant predictors of both BI and QOL, emphasizing the importance of developmental and functional factors in shaping these outcomes. Notably, fitness- and health-related aspects of BI had a stronger association with QOL than appearance-related aspects, suggesting that interventions should focus more on promoting physical health and fitness rather than appearance enhancement. This study provides valuable preliminary data that can inform the development of educational and healthcare interventions to support the holistic growth of adolescents with physical disabilities and sets the stage for further research into the factors influencing their BI and QOL.

## Figures and Tables

**Table 1:** Demographic characteristics of participants (N = 107).

Variable		N	%
Gender	Male	54	50.5
	Female	53	49.5
School level	Elementary school	22	20.6
	Middle school	45	42.1
	High school	40	37.4
Type of disability	Cerebral palsy	52	48.6
	Others	55	51.4
Activities of daily living	Mild	38	35.5
	Moderate	42	39.3
	Severe	27	25.2

**Table 2:** Scores of body image and quality of life of participants (N = 107).

Variable	*Mean*	*SD*	Cronbach’s α
Body image	2.96	0.39	0.87
Appearance evaluation	2.61	0.61	0.76
Appearance orientation	2.70	0.61	0.82
Fitness evaluation	3.03	0.93	0.82
Fitness orientation	3.02	0.70	0.89
Health evaluation	3.49	0.67	0.73
Health orientation	3.26	0.64	0.66
Quality of life	3.51	0.50	0.90
Physical	3.46	0.55	0.63
Psychological	3.42	0.63	0.73
Social relationships	3.61	0.91	0.87
Environment	3.60	0.59	0.80

**Table 3:** Differences in body image according to demographic variables of participants (N = 107).

Variable	Appearance Evaluation	Appearance Orientation	Fitness Evaluation	Fitness Orientation	Health Evaluation	Health Orientation	Body Image
	*M ± SD*	*M ± SD*	*M ± SD*	*M ± SD*	*M ± SD*	*M ± SD*	*M ± SD*
Gender							
Male	2.64 ± 0.73	2.57 ± 0.73	3.23 ± 0.95	3.21 ± 0.66	3.52 ± 0.67	3.20 ± 0.70	2.99 ± 0.47
Female	2.58 ± 0.48	2.84 ± 0.43	2.84 ± 0.87	2.82 ± 0.69	3.45 ± 0.69	3.31 ± 0.57	2.94 ± 0.31
*t*	0.51	−2.40 *	2.23 *	3.04 **	0.53	−0.89	0.68
Effect size	0.10	−0.46	0.43	0.59	0.10	−0.17	0.13
School level							
Elementary school (a)	2.52 ± 0.57	2.80 ± 0.72	3.55 ± 0.90	3.08 ± 0.63	3.56 ± 0.57	3.23 ± 0.89	3.01 ± 0.42
Middle school (b)	2.73 ± 0.69	2.71 ± 0.60	3.04 ± 0.88	3.24 ± 0.78	3.33 ± 0.61	3.19 ± 0.56	3.01 ± 0.47
High school (c)	2.52 ± 0.53	2.64 ± 0.56	2.75 ± 0.89	2.74 ± 0.54	3.63 ± 0.77	3.35 ± 0.57	2.88 ± 0.27
*F*	1.48	0.50	5.69 **	6.02 **	2.24	0.73	1.51
Post hoc			a > c	b > c			
Effect size	0.03	0.01	0.10	0.10	0.04	0.01	0.03
Type of disability							
Cerebral palsy	2.67 ± 0.65	2.67 ± 0.58	2.79 ± 0.82	2.96 ± 0.75	3.43 ± 0.69	3.32 ± 0.67	2.94 ± 0.42
Others	2.55 ± 0.58	2.74 ± 0.64	3.27 ± 0.97	3.07 ± 0.64	3.55 ± 0.66	3.19 ± 0.61	2.98 ± 0.37
*t*	0.98	−0.63	−2.75 **	−0.81	−0.91	1.07	−0.58
Effect size	0.19	−0.12	−0.53	−0.16	−0.18	0.21	−0.11
ADLs							
Mild (a)	2.66 ± 0.66	2.82 ± 0.75	3.25 ± 0.80	3.19 ± 0.61	3.29 ± 0.60	3.34 ± 0.59	3.05 ± 0.44
Moderate (b)	2.46 ± 0.36	2.76 ± 0.55	3.30 ± 0.80	3.11 ±.62	3.60 ± 0.76	3.25 ± 0.67	3.00 ± 0.31
Severe (c)	2.76 ± 0.81	2.45 ± 0.38	2.31 ± 0.92	2.63 ± 0.80	3.59 ± 0.57	3.14 ± 0.65	2.77 ± 0.39
*F*	2.16	3.32 *	13.76 ***	6.15 **	2.74	0.82	4.46 *
Post hoc		a > c	a, b > c	a, b > c			a > c
Effect size	0.04	0.06	0.21	0.11	0.05	0.02	0.08

Note: ADLs = activities of daily living, post hoc = Scheffé’s tests, * *p* < 0.05, ** *p* < 0.01, *** *p* < 0.001.

**Table 4:** Differences in quality of life according to demographic variables of participants (N = 107).

Variable	Physical	Psychological	Social Relationships	Environment	Quality of Life
	*M ± SD*	*M ± SD*	*M ± SD*	*M ± SD*	*M ± SD*
Gender					
Male	3.45 ± 0.66	3.49 ± 0.68	3.66 ± 0.80	3.63 ± 0.62	3.54 ± 0.57
Female	3.47 ± 0.42	3.35 ± 0.58	3.56 ± 1.01	3.56 ± 0.57	3.48 ± 0.44
*t*	−0.21	1.21	0.57	0.59	0.69
Effect size	−0.04	0.23	0.11	0.12	0.13
School level					
Elementary school (a)	3.66 ± 0.40	3.32 ± 0.78	3.76 ± 0.90	3.78 ± 0.69	3.64 ± 0.55
Middle school (b)	3.47 ± 0.57	3.61 ± 0.62	3.74 ± 0.77	3.52 ± 0.56	3.54 ± 0.53
High school (c)	3.33 ± 0.58	3.26 ± 0.50	3.38 ± 1.03	3.59 ± 0.57	3.40 ± 0.44
*F*	2.73	3.93 *	2.03	1.52	1.81
Post hoc		b > c			
Effect size	0.05	0.07	0.04	0.03	0.03
Type of disability					
Cerebral palsy	3.41 ± 0.57	3.48 ± 0.57	3.51 ± 0.81	3.65 ± 0.57	3.52 ± 0.45
Others	3.50 ± 0.54	3.36 ± 0.69	3.71 ± 1.00	3.55 ± 0.62	3.50 ± 0.56
*t*	−0.89	0.96	−1.16	0.90	0.20
Effect size	−0.17	0.19	−0.22	0.17	−0.34
ADLs					
Mild (a)	3.40 ± 0.65	3.41 ± 0.61	3.22 ± 1.07	3.47 ± 0.58	3.41 ± 0.58
Moderate (b)	3.55 ± 0.46	3.25 ± 0.62	3.86 ± 0.71	3.75 ± 0.66	3.57 ± 0.47
Severe (c)	3.40 ± 0.54	3.70 ± 0.61	3.78 ± 0.77	3.54 ± 0.46	3.56 ± 0.44
*F*	0.99	4.60 *	6.03 **	2.44	1.26
Post hoc		c > b	b, c > a		
Effect size	0.02	0.08	0.10	0.05	0.02

Note: ADLs = activities of daily living, post hoc = Scheffé’s tests, * *p* < 0.05, ** *p* < 0.01.

**Table 5:** Correlations between body image and quality of life of participants (N = 107).

	Physical	Psychological	Social Relationships	Environment
Appearance evaluation	0.09	0.42 ***	0.24 *	−0.14
Appearance orientation	−0.06	−0.13	0.03	−0.10
Fitness evaluation	0.39 ***	0.22 *	0.21 *	0.35 ***
Fitness orientation	0.25 *	0.18	0.09	0.24 *
Health evaluation	0.39 ***	0.06	0.38 ***	0.34 ***
Health orientation	0.31 **	0.25 *	0.14	0.27 **

Note: * *p* < 0.05, ** *p* < 0.01, *** *p* < 0.001.

## Data Availability

The data presented in this study are not publicly available but are available from the corresponding author on reasonable request.
